# Evaluation of the Association between Arsenic and Diabetes: A National Toxicology Program Workshop Review

**DOI:** 10.1289/ehp.1104579

**Published:** 2012-08-10

**Authors:** Elizabeth A. Maull, Habibul Ahsan, Joshua Edwards, Matthew P. Longnecker, Ana Navas-Acien, Jingbo Pi, Ellen K. Silbergeld, Miroslav Styblo, Chin-Hsiao Tseng, Kristina A. Thayer, Dana Loomis

**Affiliations:** 1 Biomolecular Screening Branch, Division of the National Toxicology Program, National Institute of Environmental Sciences (NIEHS), National Institutes of Health (NIH), Department of Health and Human Services (DHHS), Research Triangle Park, North Carolina, USA; 2 Departments of Health Studies, Medicine, and Human Genetics, University of Chicago Cancer Research Center, Chicago, Illinois, USA; 3 Department of Pharmacology, Midwestern University, Downers Grove, Illinois, USA; 4 Epidemiology Branch, NIEHS, NIH, DHHS, Research Triangle Park, North Carolina, USA; 5 Department of Environmental Health Sciences, and; 6 Department of Epidemiology, Johns Hopkins Bloomberg School of Public Health, Baltimore, Maryland, USA; 7 Institute for Chemical Safety Sciences, The Hamner Institutes for Health Sciences, Research Triangle Park, North Carolina, USA; 8 Department of Nutrition, University of North Carolina at Chapel Hill, Chapel Hill, North Carolina, USA; 9 National Taiwan University College of Medicine, Taipei, Taiwan; 10 Department of Internal Medicine, National Taiwan University Hospital, Taipei, Taiwan; 11 Office of Health Assessment and Translation, Division of the National Toxicology Program, NIEHS, NIH, DHHS, Research Triangle Park, North Carolina, USA; 12 Department of Epidemiology, University of Nebraska Medical Center College of Public Health, Omaha, Nebraska, USA

**Keywords:** animal, arsenic toxicity, cell line, chemically induced/epidemiology, cultured cell, diabetes, environmental epidemiology, glucose, insulin, metabolism, obesity

## Abstract

Background: Diabetes affects an estimated 346 million persons globally, and total deaths from diabetes are projected to increase > 50% in the next decade. Understanding the role of environmental chemicals in the development or progression of diabetes is an emerging issue in environmental health. In 2011, the National Toxicology Program (NTP) organized a workshop to assess the literature for evidence of associations between certain chemicals, including inorganic arsenic, and diabetes and/or obesity to help develop a focused research agenda. This review is derived from discussions at that workshop.

Objectives: Our objectives were to assess the consistency, strength/weaknesses, and biological plausibility of findings in the scientific literature regarding arsenic and diabetes and to identify data gaps and areas for future evaluation or research. The extent of the existing literature was insufficient to consider obesity as an outcome.

Data Sources, Extraction, and Synthesis: Studies related to arsenic and diabetes or obesity were identified through PubMed and supplemented with relevant studies identified by reviewing the reference lists in the primary literature or review articles.

Conclusions: Existing human data provide limited to sufficient support for an association between arsenic and diabetes in populations with relatively high exposure levels (≥ 150 µg arsenic/L in drinking water). The evidence is insufficient to conclude that arsenic is associated with diabetes in lower exposure (< 150 µg arsenic/L drinking water), although recent studies with better measures of outcome and exposure support an association. The animal literature as a whole was inconclusive; however, studies using better measures of diabetes-relevant end points support a link between arsenic and diabetes.

Diabetes, both type 1 and type 2 (T2D), is a major threat to public health in the United States and abroad [Centers for Disease Control and Prevention (CDC) 2011; Danaei et al. 2011; World Health Organization (WHO) 2011]. Based on data from the 2005–2008 National Health and Nutrition Examination Survey (NHANES), approximately 25.6 million, or 11.3%, of all persons in the United States ≥ 20 years of age have diagnosed or undiagnosed diabetes, resulting in estimated direct medical costs and indirect costs (disability, work loss, premature death) of $174 billion in 2007 alone (CDC 2011). Another 35% of persons ≥ 20 years of age are prediabetic (American Diabetes Association 2011; Knowler et al. 2002). Diabetes is now being diagnosed in individuals earlier in life [National Institute of Diabetes and Digestive and Kidney Diseases (NIDDK) 2011]. Although approximately 70% of T2D is attributed to being overweight or obese (Eyre et al. 2004), 30% of T2D cases are not attributable to obesity. Given the number of persons impacted by T2D—346 million worldwide (WHO 2011)—and its long-term consequences in terms of morbidity, mortality, and economic costs, there is considerable interest in understanding the contribution of nontraditional risk factors to the diabetes epidemic, including environmental chemicals.

Research addressing the role of environmental chemicals in diabetes manifestation has rapidly expanded. The February 2011 *Diabetes Strategic Plan* (NIDDK 2011) acknowledged the need to understand the role of environmental exposures as part of future research and prevention strategies. To help develop such a research strategy, the National Institute of Environmental Health Sciences/National Toxicology Program (NIEHS/NTP) organized a state-of-the-science workshop in January 2011 entitled Role of Environmental Chemicals in the Development of Diabetes and Obesity (NTP 2011b). The objective of this workshop was to assess the literature for evidence of associations between diabetes and/or obesity with chemicals, including arsenic, persistent organic pollutants, maternal smoking during pregnancy, bisphenol A, phthalates and organotins, and nonpersistent pesticides (Thayer et al. 2012). This report is derived from discussions on arsenic that occurred at the workshop.

The arsenic evaluation focused on diabetes only, as studies have not assessed obesity as a primary health outcome. Our review focused on the *a*) consistency, strength/weaknesses, and biological plausibility of findings, *b*) identification of the most useful and relevant end points in experimental animals and mechanistic studies, and *c*) identification of data gaps and areas for future evaluation/research.

## Identification of Relevant Studies

A PubMed (National Library of Medicine, Washington, DC, USA) search strategy, first conducted on 24 August 2009 and then run weekly until 15 December 2010, was developed to identify human, animal, and mechanistic studies (including *in vitro* assays) on arsenic exposures related to diabetes and obesity using MeSH (Medical Subject Headings)-based and keyword strategies [for search terms, see Supplemental Material, p. 2 (http://dx.doi.org/10.1289/ehp.1104579)]. A total of 108 publications were identified from the search, and 38 of those presented original data concerning both arsenic exposure and diabetes (or diabetes-related end points and/or mechanisms) and were considered relevant (see Supplemental Material, Figure S1). An additional 38 studies were identified during the course of the initial primary literature review and discussions with workshop participants, including two studies that had been submitted but not yet accepted for publication (Del Razo et al. 2011; Paul et al. 2011), for a total of 76 studies considered as the final primary literature. Two of these studies included more than one type of data, human and animal (Wang et al. 2009) or animal and *in vitro* (Yen et al. 2007).

One goal of the review was to assess the scientific literature using the descriptors “sufficient,” “limited,” or “insufficient” to classify existing evidence, with NTP definitions utilized for the NTP *Report on Carcinogens* as a framework for “sufficient” and “limited” (NTP 2011a). “Sufficient” evidence for human studies indicates a causal relationship between exposure to the agent, substance, or mixture and an outcome based on evidence of a dose–response and other characteristics such as consistency and coherence among different studies, adequate control for other covariates, biological plausibility, and adequate identification of sources of potential bias. “Limited” evidence indicates that causal interpretation is credible but that alternative explanations, such as chance, bias, or confounding factors could not adequately be excluded. The term “insufficient” is used when there is low confidence in the body of evidence to reach a conclusion on the association between exposure to a substance and health outcome(s) or when no data are available.

Epidemiological studies were classified as *a*) occupational studies; *b*) population-based studies in areas with relatively high environmental arsenic exposure (≥ 150 µg/L in drinking water); *c*) population-based studies in areas with lower arsenic exposure (< 150 µg/L in drinking water) excluding NHANES studies; and *d*) NHANES studies. The cut points used for drinking-water arsenic were selected to distinguish between high-exposure studies in areas with unusually high exposures via drinking water (e.g., in areas of Taiwan and Bangladesh) and low-to-moderate exposure studies.

## Epidemiological Studies

The first epidemiological studies reporting associations between arsenic and diabetes were published in the mid-1990s. These early studies were conducted in populations exposed to high levels of arsenic in drinking water in Taiwan and Bangladesh or were occupational studies of copper smelter and glass workers in the United States and Europe exposed to dust and particulates as distinct from water. Previous reviews of studies published before 2008 concluded that arsenic exposure was most consistently associated with diabetes in areas of Taiwan and Bangladesh with high arsenic contamination of drinking water in the past, whereas results from occupational studies and studies of populations with low-to-moderate arsenic levels in drinking water were inconsistent (Chen et al. 2007; European Food Safety Authority 2009; Longnecker and Daniels 2001; Navas-Acien et al. 2006; Tseng et al. 2002). More than 10 new epidemiological studies of arsenic exposure and diabetes have been published since 2007.

Detailed descriptions of all of the epidemiological studies considered for the review can be found in the technical literature review document prepared for the NTP workshop (NTP 2011b). Eight occupational studies also were considered as part of the review [see Supplemental Material, Table S1 (http://dx.doi.org/10.1289/ehp.1104579)] but are not considered further in this report because of concerns about diabetes assessment, exposure misclassification, and limited power. Most of the occupational studies ascertained diabetes based on death certificates, which are well known to have low sensitivity and specificity for diabetes (Cheng et al. 2008). In addition, arsenic exposure was determined based on job title, and with one exception (Lubin et al. 2000) the sample size or number of individuals with diabetes was small. This assessment of the occupational studies is consistent with other reviews of arsenic (Longnecker and Daniels 2001; Navas-Acien et al. 2006).

*Environmental exposure settings.* Of the 27 eligible nonoccupational publications that met our inclusion criteria, 9 were classified as high exposure (Table 1), 15 were classified as non-NHANES studies with low-to-moderate exposure (Table 2), 1 was classified as both low and high exposure (Chen et al. 2010), and 4 were classified as analyses of NHANES data (Table 2). Two high-exposure studies used a prospective design (Tseng et al. 2000a, 2000b), and the rest were cross-sectional (*n* = 12, excluding the NHANES studies), case–control (*n* = 5), or retrospective (*n* = 4). Three studies did not report risk estimates for diabetes, but compared the levels of arsenic in persons with diabetes (diabetics) and nondiabetics (Afridi et al. 2008; Kolachi et al. 2010; Serdar et al. 2009).

**Table 1:** Association between arsenic and diabetes in areas of relatively high exposure (≥ 150 µg/L drinking water).

Reference (study design)	Location, subjects	Diabetes diagnosis	Main findinga,b	Exposurec	Factors considered in analysis
Chen et al. 2010 (cross-sectional)	Bangladesh (Araihazar) HEALS, n = 11,319 ♂♀	Self-report prior to baseline	1.11 (95% CI: 0.73, 1.69) adjOR	176.2–864 (Q5) vs. 0.1–8 (Q1) µg As/L drinking water, CEI Cohort: 0.1–864 µg As/L	Age, sex, BMI, smoking status, educational attainment
Lai et al. 1994 (cross-sectional)	Taiwan (southern) As-endemic region, n = 891 ♂♀	Self-report, OGTT, treatment history	10.05 (95% CI: 1.3, 77.9) adjOR	≥ 15 vs. 0 ppm-year drinking water, CEI Cohort: 780 (700–930) µg As/L; median (range) concentrations in artesian wellsd	Age, sex, BMI, physical activity
Nabi et al. 2005e (case–control)	Bangladesh (Chapainowabganj) arsenicosis cases, n = 235 ♂♀	Glucose, blood	2.95 (95% CI: 0.954, 9.279) OR	218.1 vs. 11.3 (mean) µg As/L drinking water Cohort: 218.1 (3–875) µg As/L; mean (range)	Unadjusted
Rahman et al. 1998f (cross-sectional)	Bangladesh (Dhaka) keratosis cases, n = 1,107 ♂♀	Self-report, OGTT, glucosuria	5.2 (95% CI: 2.5, 10.5) adjPR	Keratosis vs. non-keratosis Cohort: < 10–2,100 µg As/L	Age
Rahman et al. 1999f (cross-sectional)	Bangladesh (multisite) with skin lesions, n = 430 ♂♀	Glucosuria	2.9 (95% CI: 1.6, 5.2) adjPR	> 10 vs. < 1 mg-year As/L drinking water, CEI Cohort: < 500 to > 1,000 µg As/L drinking water	Age, sex, BMI
Tsai et al. 1999e (retrospective)	Taiwan (Chiayi County) Blackfoot region, n = 19,536 deaths ♂♀	Death certificate	1.46 (95% CI: 1.28, 1.67) SMR	Blackfoot endemic region vs. national reference Cohort: 780 (250–1,140) µg As/L; median (range)	Age, sex
Tseng et al. 2000a, 2000b (prospective)	Taiwan (southwestern) agricultural and aquacultural regions, n = 446 ♂♀	Fasting blood glucose, OGTT	2.1 (95% CI: 1.1, 4.2) RR	≥ 17 vs. < 17 mg/L-year As (drinking water, CEI) Cohort: 700–930 µg As/L; range of median concentration in artesian wells	Age, sex, BMI
Wang SL et al. 2003g (cross-sectional)	Taiwan (southwestern) As-endemic region, n = 706,314 ♂♀	Insurance claims	2.69 (95% CI: 2.65, 2.73) adjOR	Endemic vs. non-endemic region Cohort: 780 (350–1,140) µg As/L; median (range)d	Age, sex
Abbreviations: adjOR, adjusted odds ratio; adjPR, adjusted prevalence ratio; As, arsenic; BMI, body mass index; CEI, cumulative exposure index; HEALS, Health Effects of Arsenic Longitudinal Study; mg-year, milligram year; OGTT, oral glucose tolerance test; OR, odds ratio; Q, quintile; RR, relative risk; SMR, standardized mortality ratio. aIdentification of main findings was based on the following strategy: for studies that did not report a significant association between arsenic exposure and a health outcome at any exposure level, the main summary finding was based on the highest exposure group compared to the referent group (e.g., 4th quartile vs. 1st quartile). When a study reported a significant association between arsenic exposure and a health outcome, the main finding was based on lowest exposure group where a statistically significant association was observed (e.g., 3rd quartile vs. 1st quartile). bUnless specified, relative risk estimates are crude estimates. cMedian or mean and range of As concentration in drinking water for the cohort is included when reported. dArsenic drinking-water concentrations were taken from other publications based on same populations. eCalculated by entering data presented in publication into OpenEpi software (Dean et al. 2011). fAlthough the arsenic water concentrations are expressed in units of mg/L, the value is supposed to represent the “approximate time-weighted mean arsenic exposure levels that were calculated over the lifetime of each subject as ∑j(ajcj/∑jaj, where aj is the number of years a well with arsenic concentration cj was used, assuming that the current levels of arsenic in the well water were also representative of the past source.” gThere appears to be an error in the number of persons included in the “non-endemic” area category based on the ns provided in Table 1 of Wang et al. 2003.										

**Table 2:** Association between arsenic and diabetes-related measures in areas of relatively low-to-moderate exposures (< 150 µg/L drinking water) and NHANES.

Reference (study design)	Location, subjects	Diabetes diagnosis	Main findinga,b	Exposurec	Factors considered in analysis
Afridi et al. 2008d (cross-sectional)	Pakistan (Hyderabad), n = 225 ♂ (nonsmokers) and n = 209 ♂ (smokers)	Self-report	↑ Urinary As in nonsmoking diabetics	Nonsmokers: 5.59 (diabetics) vs. 4.7 (nondiabetics) µg As/L, mean (urine) Smokers: 7.27 (diabetics) vs. 5.41 (nondiabetics) µg As/L Cohort: drinking-water concentrations not reported	Unadjusted
Chen et al. 2010 (cross-sectional)	Bangladesh (Araihazar), HEALS, n = 11,319 ♂♀	Self-report prior to baseline	1.24 (95% CI: 0.82, 1.87) adjOR	41–92 (Q3) vs. 0.1–8 (Q1) µg As/L drinking water, CEI Cohort: 0.1–864 µg As/L	Age, sex, BMI, smoking status, educational attainment; (similar results obtained when model only adjusted for age, sex, BMI)
Coronado-González et al. 2007 (case–control)	Mexico (Coahuila) As-endemic region, n = 400 ♂♀	Fasting blood glucose, treatment history	2.84 (95% CI: 1.64, 4.92) adjOR	> 104 (T3) vs. < 63.5 (T1) µg As/g creatinine (urine) Cohort: 20–400 µg As/L drinking water reported in other studies of the region	Age, sex, hypertension, family history, obesity, serum lipids
Del Razo et al. 2011 (cross-sectional)	Mexico (Zimapan and Lagunera) As-endemic region, n = 258 ♂♀	Fasting blood glucose	1.13 (95% CI: 1.05, 1.22) adjOR per 10 µg As/L ↑	Cohort: 42.9 mean (3–215, range) µg As/L (current drinking water)	Age, sex, obesity, hypertension
Ettinger et al. 2009 (cross-sectional)	USA (Tar Creek, OK), n = 456 pregnant ♀	Impaired glucose tolerance (OGTT)	2.79 (95% CI: 1.13, 6.87) adjOR	2–24 (Q4) vs. 0.2–0.9 (Q1) µg As/L (blood) Cohort: reported from other studies that at least 25% of samples in region have > 10 µg As/L drinking water	Age, pre-pregnancy BMI, ethnicity/race, Medicaid use, married or living with partner
Kolachi et al. 2010 (case–control)	Pakistan (Hyderabad) diabetes, n = 144 ♀	IDDM (fasting blood glucose, OGTT)	↑ Urine As in diabetics	4.13 (diabetics) vs. 1.48 (nondiabetics) µg As/L, mean (urine) Cohort: drinking-water concentrations not reported	Unadjusted
Lewis et al. 1999 (retrospective)	USA (7 communities in Millard County, UT), n = 961 ♀ deaths; n = 1,242 ♂ deaths	Death certificate	♀: 1.23 (95% CI: 0.86, 1.71) SMR ♂: 0.79 (95% CI: 0.48, 1.22) SMR	Millard vs. state Cohort: 14–166 µg (3.5–620) µg As/L, range of median well-water concentrations between 1976–1997 (overall range)	Sex, race
Meliker et al. 2007 (retrospective)	USA (6 counties in southeastern MI), n = 41,282 ♂ deaths; n = 38,722 ♀ deaths	Death certificate	♂: 1.28 (95% CI: 1.18, 1.37) SMR ♀: 1.27 (95% CI: 1.19, 1.35) SMR	6 counties vs. state µg As/L (drinking water) Cohort: 7.58 (1.27–11.98) µg As/L, population weighted median across 6 counties (range)	Sex, race
Ruiz-Navarro et al. 1998e (case–control)	Spain (Motril) hospital patients, n = 87 ♂♀	Not reported	0.87 (95% CI: 0.5, 1.53) RR	75th vs. 25th percentile µg As/L (urine) Cohort: drinking-water concentrations not reported	Unadjusted
Serdar et al. 2009 (cross-sectional)	Turkey (Ankara), n = 87 diabetes clinic patients	Treatment history	↔ Plasma As in diabetics vs. controls	1.22 (diabetics) vs. 0.86 (nondiabetics) µg As/L (plasma) Cohort: drinking-water concentrations not reported	Unadjusted
Tollestrup et al. 2003e (retrospective)	USA (Ruston, WA) lived near smelter as children, n = 1,074 deaths ♂♀	Death certificate	1.6 (95% CI: 0.36, 7.16) RR	Residence time within 1.6 km (1 mi): ≥ 10 years vs. < 1 year Cohort: drinking-water concentrations not reported	Unadjusted
Continued					
Table 2. Continued					
Reference (study design)	Location, subjects	Diabetes diagnosis	Main findinga,b	Exposurec	Factors considered in analysis
Wang SL et al. 2007 (cross-sectional)	Taiwan (central) industrial region, n = 660 ♂♀	Metabolic syndrome (fasting blood glucose, triglycerides, HDL, blood pressure, BMI)	2.35 (95% CI: 1.02, 5.43) adjOR	“High” vs. “low” µg As/g hair Cohort: 2002–2005 groundwater concentrations for area ranged from ~6 to ~15 µg As/L	Age, sex, occupation, lifestyle factors (alcohol, betel nut chewing, smoking, groundwater use)
Wang JP et al. 2009f (cross-sectional)	China (Xinjiang region) As-endemic region, n = 235 ♂♀	Hospital records, exam	1.098 (95% CI: 0.98, 1.231) RR	21–272 (range) vs. 16–38 (range) µg As/L (drinking water) Cohort: 16–272 µg As/L drinking water	Unadjusted
Ward and Pim 1984f (case–control)	U.K. (Oxford, England) diabetes clinic patients, n = 117 ♂♀	Not reported	1.09 (95% CI: 0.79, 1.49) RR	75th vs. 25th percentile µg As/mL (plasma) Cohort: drinking-water concentrations not reported	Unadjusted
Zierold et al. 2004g (cross-sectional)	U.S. (WI) well-water testing program, n = 1,185 ♂♀	Self-report	1.02 (95% CI: 0.49, 2.15) adjOR	> 10 vs. < 2 µg As/L (well-water) Cohort: 2 (0–2,389) µg As/L; median (range)	Age, sex, BMI, smoking
Navas-Acien et al. 2008 (cross-sectional)	U.S. (NHANES 2003–2004) ≥ 20 years, n = 788 ♂♀	Fasting blood glucose, self-report, medication	3.58 (95% CI: 1.18, 10.83) adjOR	18 (≥ 80th) vs. 3.5 (≤ 20th percentile) µg As/L (urine)	Sex, age, race, urine creatinine, education, BMI, serum cotinine level, hypertension medication, urine arsenobetaine, blood mercury levels
Navas-Acien et al. 2009a (cross-sectional)	U.S. (NHANES 2003–2006) ≥ 20 years, n = 1,279 ♂♀ with arsenobetaine < LOD	Fasting blood glucose, self-report, medication	2.60 (95% CI: 1.12, 6.03) adjOR	7.4 (80th) vs. 1.6 (20th percentile) µg As/L (urine)	Sex, age, race, urine creatinine, education, BMI, serum cotinine level, hypertension medication, blood mercury levels
Steinmaus et al. 2009a (cross-sectional)	U.S. (NHANES 2003–2004) ≥ 20 years, n = 795 ♂♀	Fasting blood glucose, self-report, medication	1.15 (95% CI: 0.53, 2.50) adjOR	12 (≥ 80th) vs. 2.7 ( ≤ 20th percentile) µg As/L (urine, not adjusted for creatinine) [urine As = total As – (arsenobetaine + arsenocholine)]	Sex, age, ethnicity, education, BMI, serum cotinine, urine creatinine, current use of hypertension medications
Steinmaus et al. 2009b (cross-sectional)	U.S. (NHANES 2003–2006) ≥ 20 years, n = ~1,280 ♂♀ with arsenobetaine < LOD	Fasting blood glucose, self-report, medication	1.03 (95% CI: 0.38, 2.80) adjOR	≥ 80th vs. ≤ 20th percentile µg As/L (urine, not adjusted for creatinine)	Sex, age, race, BMI
Abbreviations: adjOR, adjusted odds ratio; adjPR, adjusted prevalence ratio; As, arsenic; BMI, body mass index; CEI, cumulative exposure index; HDL, high density lipoproteins; IDDM, insulin dependent diabetes mellitus; LOD, level of detection; MI, Michigan; OK, Oklahoma; Q, quintile; RR, relative risk; SMR, standardized mortality ratio; T, tertile; UT, Utah; WA, Washington. aIdentification of main findings was based on the following strategy: For studies that did not report a significant association between arsenic exposure and a health outcome at any exposure level, the main summary finding was based on the highest exposure group compared to the referent group (e.g., 4th quartile vs. 1st quartile). When a study reported a significant association between arsenic exposure and a health outcome, the main finding was based on lowest exposure group where a statistically significant association was observed (e.g., 3rd quartile vs. 1st quartile). bUnless specified, relative risk estimates are crude estimates. cMedian or mean and range of As concentration in drinking water included, when provided in the primary literature. dThe standard deviations presented in the study may be SEs. eRelative risk and 95% confidence interval as estimated by Navas-Acien et al. (2006). fCalculated by entering data presented in publication into OpenEpi software (Dean et al. 2011). gNumber of cases were not reported in original study, but were reported by Navas-Acien et al. (2006).					

Diabetes ascertainment differed among studies. Four studies used death certificates to ascertain diabetes (Lewis et al. 1999; Meliker et al. 2007; Tollestrup et al. 2003; Tsai et al. 1999) and three others used exclusively self-reported history of diabetes (Afridi et al. 2008; Chen et al. 2010; Zierold et al. 2004). Two studies used diagnosis of diabetes but did not report the basis of diabetes diagnosis (Ruiz-Navarro et al. 1998; Ward and Pim 1984). Seven studies, generally those conducted more recently, incorporated diagnostic indicators such as fasting glucose or oral glucose tolerance test (OGTT) results (Coronado-González et al. 2007; Del Razo et al. 2011; Ettinger et al. 2009; Kolachi et al. 2010; Rahman et al. 1998; Tseng et al. 2000b; Wang et al. 2007). Two other studies reported risk estimates for metabolic syndrome (Wang et al. 2007) and impaired glucose tolerance (Ettinger et al. 2009) rather than diabetes. Many of the studies were conducted in Bangladesh [*n* = 4 (Chen et al. 2010; Nabi et al. 2005; Rahman et al. 1998, 1999)] or Taiwan [*n* = 5 (Lai et al. 1994; Tsai et al. 1999; Tseng et al. 2000b; Wang et al. 2003, 2007)]. Other countries included the United States (Ettinger et al. 2009; Lewis et al. 1999; Meliker et al. 2007; Navas-Acien et al. 2008, 2009a; Steinmaus et al. 2009a, 2009b; Tollestrup et al. 2003; Zierold et al. 2004), Mexico (Coronado-González et al. 2007; Del Razo et al. 2011), Pakistan (Afridi et al. 2008; Kolachi et al. 2010), Turkey (Serdar et al. 2009), Spain (Ruiz-Navarro et al. 1998), China (Wang et al. 2009), and the United Kingdom (Ward and Pim 1984).

Measures of exposure are highly variable between these studies, ranging from areawide exposure estimates based on measurement of arsenic from drinking-water sources to individual-level exposure estimates based on detailed water consumption history, work history, or actual biomarkers of exposure. These variations in study design constitute irreducible sources of heterogeneity and present interpretive challenges in evaluating the results observed in this collection of studies. Specifically, exposure was assessed by arsenic concentrations in drinking water within a geographic area (Del Razo et al. 2011; Meliker et al. 2007; Zierold et al. 2004), as cumulative exposure index based on residence time × average drinking-water level (Chen et al. 2010; Lai et al. 1994; Lewis et al. 1999; Rahman et al. 1999; Tseng et al. 2000b), residence time in an arsenicosis-endemic region (Tollestrup et al. 2003; Tsai et al. 1999; Wang et al. 2003) or presence or absence of arsenicosis or keratosis as a surrogate for long-term exposure to arsenic (Nabi et al. 2005; Rahman et al. 1998) or by biomarkers including blood/plasma arsenic levels (Ettinger et al. 2009; Serdar et al. 2009; Ward and Pim 1984) and arsenic concentration in urine (Coronado-González et al. 2007; Navas-Acien et al. 2008, 2009a; Ruiz-Navarro et al. 1998; Steinmaus et al. 2009a, 2009b; Wang et al. 2009) or hair (Afridi et al. 2008; Kolachi et al. 2010; Wang et al. 2007). Three studies did not report risk estimates, but compared the levels of arsenic in diabetics and nondiabetics. Afridi et al. (2008) measured higher levels of arsenic in the hair, blood, and urine of 196 diabetics participating in a study that included a total of 434 men from Hyderabad, Pakistan. Higher arsenic urine, blood, and hair levels were also found in diabetics compared to nondiabetics in another study conducted in Pakistan by Kolachi et al. (2010). Levels of hair arsenic were significantly higher in a group of 76 new mothers with insulin-dependent diabetes compared to a group of 68 nondiabetic mothers, although hair is not considered the preferred matrix for arsenic [National Research Council (NRC) 1999]. Serdar et al. (2009) did not detect any statistically significant differences in plasma arsenic in diabetes cases (*n* = 31, mean ± SD = 1.22 ± 0.57 µg/L) compared to controls [*n* = 22; mean (range) = 0.86 (0.64–1.59 µg/L)] in a study based in Turkey, although this study may have been underpowered to detect differences.

*Environmental exposure, high arsenic areas (≥ 150 µg/L drinking water)*. Table 1 summarizes the high-arsenic environmental exposure studies from Bangladesh (Chen et al. 2010; Nabi et al. 2005; Rahman et al. 1998, 1999) and Taiwan (Lai et al. 1994; Tsai et al. 1999; Tseng et al. 2000a, 2000b; Wang et al. 2003). There is limited to sufficient evidence for an association between arsenic and diabetes in populations from high-arsenic areas, primarily occurring in Bangladesh or Taiwan. Support for an association was strongest in studies where arsenic drinking-water levels were > 500 µg/L (Lai et al. 1994; Nabi et al. 2005; Rahman et al. 1998, 1999; Tsai et al. 1999; Tseng et al. 2000b; Wang et al. 2003). Eight of the nine studies conducted in Taiwan or Bangladesh reported positive associations between arsenic and diabetes (Table 1) (Lai et al. 1994; Nabi et al. 2005; Rahman et al. 1998, 1999; Tsai et al. 1999; Tseng et al. 2000a, 2000b; Wang et al. 2003). The only prospective study within this group also reported a positive association [adjusted relative risk (RR) = 2.1 (95% CI: 1.1, 4.2)] for development of diabetes over a 4-year follow-up period among individuals with ≥ 17 mg/L-years compared with < 17 mg/L-years cumulative arsenic exposure (Tseng et al. 2000b). Those studies relying on clinically accepted measures of disease (e.g., fasting blood glucose, OGGT) (Lai et al. 1994; Rahman et al. 1998; Tseng et al. 2000a, 2000b) reported risk estimates ranging from 2.1 (RR; 95% CI: 1.1, 4.2) to 10.05 [adjusted odds ratio (adjOR); 95% CI: 1.3, 77.9]. Some of the studies might not be completely independent if they were surveying the same population, and perhaps the same individuals. Of the studies conducted in Taiwan, several (Lai et al. 1994; Tsai et al. 1999; Tseng et al. 2000b; Wang et al. 2003) derived their study populations from the Southwestern Blackfoot or arseniasis-endemic region of Taiwan. Furthermore, several papers specifically included the village of Pu-Tai (Lai et al. 1994; Tseng et al. 2000a, 2000b). Data presented by Tseng et al. (2000a, 2000b) represent a follow-up to the Lai et al. (1994) study and therefore likely included many of the same participants. Studies conducted in Bangladesh have focused on the same geographical area for their exposed populations: Dhaka, Rajshahu, and Khulna Divisions (Chen et al. 2010; Nabi et al. 2005; Rahman et al. 1998, 1999). While none of the Bangladesh studies indicated that they were follow-up activities related to previous studies, participants may have overlapped.

In contrast to the relative strength and consistency of results in many of the high-exposure studies, the most recent and largest study in Bangladesh did not find any significant associations between urinary arsenic or time-weighted average water arsenic and self-reported diabetes, glucosuria, or hemoglobin A1c (HbA1c) levels in a population-based cross-sectional study of 11,319 Bangladeshi men and women participating in the Health Effects of Arsenic Longitudinal Study (HEALS) (Chen et al. 2010). Diagnosis of diabetes was based on self-report of physician diagnosis prior to baseline, glucosuria (excluding 90 individuals who were taking medications for diabetes), or, in a smaller subset of 2,100 participants, HbA1c. Although the Chen et al. (2010) cohort is large, statistical power was limited by the small number of diabetes cases (241 of 11,078; about 2% of the total cohort reported a diagnosis of diabetes prior to baseline, including 45 diabetes cases in the highest quintile category for time-weighted average arsenic). Nonetheless, while a number of explanations for the findings of Chen et al. (2010) exist, no definitive conclusions could be drawn regarding aspects of the study design or population (e.g., nutritional status, obesity, genetic differences) or exposure history (i.e., the relatively short duration of exposure for some study participants compared with the experiences of individuals in the arsenic-contaminated areas of Taiwan) that could explain the difference between this and the other studies.

*Environmental exposure, low-to-moderate arsenic areas*. Excluding the NHANES studies, 12 of the 15 identified epidemiologic studies reported risk estimates related to diabetes, glycemic control, or metabolic syndrome in populations under conditions of low-to-moderate arsenic exposure from drinking water (< 150 µg/L drinking water) (Table 2). Two studies (Lewis et al. 1999; Meliker et al. 2007) evaluated SMRs for each sex separately. The highest categories of drinking-water exposure in these studies were lower than the arsenic-exposed population studies in Bangladesh and Taiwan. Overall, the current literature provides insufficient evidence to conclude that arsenic is associated with diabetes at these levels of exposure. Recent studies with better measures of outcome (fasting blood glucose levels or OGTT) reported more consistent associations between arsenic and diabetes (Coronado-González et al. 2007; Del Razo et al. 2011) or impaired glucose tolerance (Ettinger 2009) within this range of exposure. Some of the differences among the studies may be due to variation in sample sizes and to differences in study populations and methods used to classify diabetes (e.g., death certificates vs. self-report or blood glucose level) or to estimate arsenic exposure (e.g., urine levels vs. drinking-water surveys).

Four publications based on analyses of data from NHANES cohorts, which are representative of the U.S. population and generally include participants with low-to-moderate exposure, were considered in our review (Navas-Acien et al. 2008, 2009a; Steinmaus et al. 2009a, 2009b). However, the results of these studies should not be considered independent because the main focus of several of the publications was to compare the methodological strategies used to assess the association between urinary arsenic and diabetes. In brief, differences in interpretation of the association between arsenic and diabetes can be reached based on different methodological approaches used to account for organic arsenic due to seafood consumption and whether to include urinary creatinine as an adjustment factor in the statistical model. Results of two of the NHANES analyses supported an association between arsenic exposure and diabetes (Navas-Acien et al. 2008, 2009a), but results based on two alternative analyses did not (Steinmaus et al. 2009a, 2009b). Differences in methodological approaches used to characterize arsenic exposure in these studies are discussed in more detail below under “Urinary arsenic.”

*Determining exposure and internal dose in studies of arsenic*. Arsenic concentrations in drinking water. Measurement of total arsenic in drinking-water supplies is often used to assess arsenic exposure, but this approach is not appropriate for research questions pertaining to individual exposures, including research concerning the effects of individual variation in arsenic metabolism on internal dose. Individual-level information on the magnitude, duration, and timing of exposure is critical, especially for estimating cumulative exposure. One alternative has been to combine historical measurements of arsenic concentrations in drinking water with self-reported residential and water-use histories. This approach usually requires an assumption that arsenic concentrations in drinking water are stable over time and that study subjects do not consume water from other sources. Support for these assumptions has been found in several study populations (Navas-Acien et al. 2009b; Ryan et al. 2000).

Arsenic levels in blood, nails, and hair. The literature review revealed a number of arsenic exposure biomarkers in need of further characterization and validation. Whole blood and plasma are emerging exposure matrices that reflect a shorter half-life (i.e., about 1 hr) compared to arsenic levels in urine (4 days) (NRC 1999). Hair and nail arsenic levels are noninvasive measures that reflect mean arsenic levels for exposures that occurred several months (for hair) to over a year (for nails) before sampling (Orloff et al. 2009). Moreover, arsenic levels in nails generally reflect exposure to inorganic arsenic and seem to be less affected by seafood arsenicals (see below). While sometimes useful, hair is not a recommended exposure matrix for arsenic (NRC 1999). One limitation of measuring arsenic in hair and nails is that arsenic speciation is difficult to conduct. Also, the time period of exposure captured by hair and nail measurements depends on the specific segments collected and analyzed. Other target tissues (e.g., urothelial cells) and buccal and saliva samples have also been suggested (Bartolotta et al. 2011; Hernández-Zavala et al. 2008; Lew et al. 2010). Although these emerging biomarkers deserve additional attention, a more expanded knowledge of toxicokinetic data and information on correlations with existing biomarkers and intake doses is needed before they are adopted for use in research.

Urinary arsenic. One of the most commonly used measures of arsenic exposure is urine. However, measurements of total urinary arsenic will not distinguish between inorganic and organic forms of arsenic unless a speciated analysis is conducted. Distinguishing between the inorganic and organic forms of arsenic is important because the inorganic forms are generally accepted as being of greater toxicological concern than the organic forms [Agency for Toxic Substances and Disease Registry (ATSDR) 2007; Vahter and Concha 2001]. The metabolism of inorganic arsenic is complex and results in a number of metabolites, including some that are chemically unstable. Inorganic arsenic occurs in two oxidation states: arsenite (AsIII) and arsenate (AsV), where the Roman numeral refers to the oxidation state. In the process of forming more water-soluble molecules, inorganic arsenic goes through alternating reduction and methylation reactions and fluctuates between oxidation states of III (regarded as more toxic) and V (less toxic) (ATSDR 2007; Vahter and Concha 2001). The general characterization of oxidation state III as less toxic than V is primarily based on acute toxicity studies, and this issue has not been adequately assessed in long-term toxicological studies.

**Figure 1:**
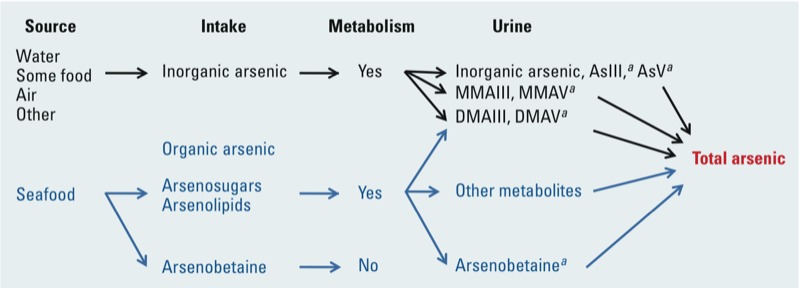
Arsenic exposure and metabolism in the human body: from source to urine (modified from Navas-Acien et al. 2009a). ***^a^***Arsenic species measured in NHANES (Caldwell et al. 2009). Two other organic forms of arsenic considered to be minor contributors to arsenic in seafood were also measured in NHANES but were detected only in a small number of urine samples: arsenocholine (1.8%) and trimethylarsine oxide (0.3%). The predominant urinary metabolite of arsenocholine in rats, mice, and rabbits is arsenobetaine (Marafante et al. 1984).

In any case, total urinary arsenic reflects the number of arsenic ions generated from all arsenic species in the urine, including inorganic arsenic (AsIII, AsV), the tri- and pentavalent methylated metabolites of inorganic arsenic [monomethylarsonite (MMAIII), dimethylarsinite (DMAIII), monomethylarsonate (MMAV), dimethylarsinate (DMAV)] and the less toxic organic arsenic compounds commonly associated with dietary exposures, particularly in seafood (mainly arsenobetaine, arsenosugars, and arsenolipids) (Caldwell et al. 2009; Navas-Acien et al. 2009b) [Figure 1; for detailed information on common forms of arsenic, see Supplemental Material, Table S2 (http://dx.doi.org/10.1289/ehp.1104579)]. Because it is currently assumed that both the inorganic forms of arsenic and their methylated metabolites may be associated with diabetes and other health risks, speciation analysis, including specification of the arsenic oxidation state, is recommended. Studies that do include a speciated analysis often do not include an oxidative state analysis to distinguish between tri- and pentavalent metabolites of inorganic arsenic. In particular, there is a need to improve the ability to measure methylated trivalent species because they are regarded as more toxic (ATSDR 2007; Vahter and Concha 2001) and concentrations may be underestimated unless the appropriate speciation analysis is conducted. Although technically challenging and not typically done, it is possible to conduct analyses of these metabolites at the point of collection.

Accounting for arsenic of seafood origin. Most human biomonitoring studies report levels of total arsenic, which includes inorganic and organic arsenic compounds and their metabolites. Depending on location and diet of the population being studied, fish and other seafood can be a significant source of exposure to specific organic forms of arsenic such as arsenobetaine, arsenosugars, and arsenolipids (Figure 1). Although they have not been evaluated as risk factors for diabetes-related end points, these complex organic arsenic compounds are generally accepted as less toxic than either inorganic arsenic or their methylated metabolites (ATSDR 2007; Vahter and Concha 2001). Inorganic arsenic as well as methylated forms in oxidation state III are highly reactive, with a high affinity for sulfhydryl groups (Vahter and Concha 2001). Therefore, failure to distinguish organoarsenicals from inorganic arsenic and metabolites of inorganic arsenic in urine may result in misclassification of exposure to the most toxicologically relevant forms of arsenic, which in turn may lead to mischaracterization of the association between urinary arsenic and diabetes. This is less of a concern when study participants are exposed to higher levels of arsenic from drinking water or proximity to an industrial or mining site with arsenic contamination because it is reasonable to assume that urinary arsenic primarily reflects exposure to inorganic arsenic in these populations. However, in studies of the general population, such as NHANES, a larger portion of urinary arsenic may represent organic arsenic, mostly due to seafood consumption (Longnecker 2009; Navas-Acien et al. 2009a; Steinmaus et al. 2009a).

How to best adjust for organic arsenicals of seafood origin is a controversial topic [for a detailed discussion, see Supplemental Material, pp. 5–7 (http://dx.doi.org/10.1289/ehp.1104579)]. Inorganic forms, arsenite and arsenate, are metabolized to their methylated forms, MMA and DMA, and eliminated in the urine. Although DMA is the major metabolite of inorganic arsenic, it is also a metabolite of the organic arsenicals, arsenosugars and arsenolipids and therefore reflects both exposures to inorganic and organic forms of arsenic of seafood origin (Figure 1). Three published strategies have been used to address this issue using NHANES data: *a*) statistically adjusting models used to estimate the association between total urinary arsenic and diabetes for markers of seafood intake, such as levels of urinary arsenobetaine and blood mercury (Navas-Acien et al. 2008), *b*) restricting the analysis to participants with very low or nondetectable levels of arsenobetaine (Navas-Acien et al. 2009a), and *c*) subtracting any organic arsenicals (i.e., arsenobetaine and arsenocholine) above detection limits from the total urinary arsenic measurement (Steinmaus et al. 2009a). These strategies led to different conclusions regarding the association between inorganic arsenic and diabetes in NHANES, with the first two approaches resulting in statistically significant associations (Navas-Acien et al. 2009a, 2011), whereas the third suggested no association (Steinmaus et al. 2009a). Subtracting arsenobetaine from total urinary arsenic does not account for exposure misclassification due to the presence of other seafood arsenicals and their metabolites, which are included in total urinary arsenic measurements but cannot be specifically accounted for because they were not measured separately in the NHANES samples. Statistical adjustment for arsenobetaine and restriction to participants with low levels of arsenobetaine control for all seafood arsenic species, not only for arsenobetaine, and have shown consistent results (Navas-Acien et al. 2009a, 2011). However, statistical adjustment may not completely eliminate bias because it mixes the effects of relevant and irrelevant exposures, and exclusion of seafood consumers from analysis may lead to selection bias in populations where seafood consumption is common. The lack of consistency of findings based on the different analytical approaches described above warrants caution in interpreting results from NHANES studies and highlights the importance of having good analytical methods to distinguish inorganic arsenic and its methylated metabolites from organic arsenicals of seafood origin.

Accounting for urine dilution. Typically, epidemiological studies that quantify exposure on the basis of spot urine measures for arsenic or other nonpersistent chemicals include adjustments for urine creatinine to account for variation in urine dilution. This may be accomplished by normalizing arsenic levels for creatinine as the exposure metric (i.e., micrograms of arsenic per gram urinary creatinine) or adjusting by using urinary arsenic as the measure of exposure (i.e., micrograms of arsenic per liter urine) but then including creatinine as a separate independent variable in the multiple regression analyses. Of the two approaches, the latter approach is recommended (Barr et al. 2005) because urinary creatinine concentrations are influenced by age, sex, health status, race/ethnicity, body mass index, fat-free mass, and time of day of collection and therefore can vary widely across individuals (Barr et al. 2005; Boeniger et al. 1993; Mahalingaiah et al. 2008). However, this strategy may not be appropriate for metals or other chemicals that compromise kidney function.

The decision on how, or whether, to adjust for urinary creatinine concentration is more complicated when the health effect under investigation can impact creatinine levels, as is the case with diabetes (Greenland 2003). Persons with diabetes tend to have lower urinary concentrations of creatinine, in part because muscle mass is reduced as a consequence of diabetes, which results in reduced creatinine excretion (Park et al. 2009). Diabetes also leads to increased glomerular filtration and increased water intake, which can cause urine to be more dilute, resulting in lower urinary creatinine concentrations (Jerums et al. 2010). Both physiological processes may lead to biased assessments on the association between urinary arsenic and diabetes, although it is not possible to predict the direction of the overall bias with confidence (i.e., systematic bias toward or away from identifying a positive association). The reasons for this are discussed in more detail in the literature review document prepared for the 2011 workshop (NTP 2011b). The situation is further complicated because arsenic exposure has also been associated with increased urine creatinine in persons living in an arsenic-endemic area of Bangladesh (Nermell et al. 2008) or participating in the HEALS study described above (Ahsan H, personal communication). Thus, if diabetes and arsenic affect creatinine production, as well as urine dilution, then adjustment for creatinine may introduce bias rather than controlling measurement error induced by urine dilution (Greenland 2003). Relative risk estimates for associations between arsenic and diabetes based on creatinine-​adjusted urine are quantitatively higher than estimates based on urinary arsenic levels that are not adjusted for creatinine (Chen et al. 2010; Steinmaus et al. 2009b). However, given the issues discussed above, it may not be possible to fully understand the potential bias with respect to clarifying the association between arsenic and diabetes. While specific gravity has been suggested as an alternative method to normalize urinary arsenic for differences in urine dilution because it appears to be less affected than creatinine by age, sex, and body size (Mahalingaiah et al. 2008; Nermell et al. 2008), its use is not recommended in studies of diabetes because it is well established that specific gravity is not an accurate method if albumin or glucose is present in the urine (Chadha et al. 2001; Voinescu et al. 2002). One approach to address concerns about creatinine adjustment is to report both raw and adjusted values. Prospective evidence, that is, measuring arsenic and creatinine at baseline and then during diabetes development over the follow-up, remains the best strategy to eliminate potential bias related to the impact of diabetes in urine creatinine concentrations (i.e., before any potential renal or metabolic effect of the disease occurs in urine creatinine concentrations).

Emerging issues related to arsenic exposure. At present, there is very little exposure or toxicity information for other types of arsenicals. Roxarsone, an arsenic-based drug fed to chicken, turkeys, and pigs for growth promotion, feed efficiency, and improved pigmentation, may be a source of dietary exposure to inorganic arsenic (Food and Drug Administration 2011; Silbergeld and Nachman 2008). Thioarsenical metabolites in urine are emerging forms of concern but are difficult to measure and their interpretation is at present unclear (Naranmandura et al. 2010; Pinyayev et al. 2011). The significance of the gut microbiome in understanding arsenic toxicity is another new issue in the field. Available data suggest the impact of microbiome metabolism of arsenic prior to absorption into the human body may be important in terms of interpreting observed differences in patterns of arsenic metabolites in addition to differences in metabolic pathways within human organs (Proctor 2011; Sun et al. 2012; Van de Wiele et al. 2010).

## Experimental Animal Studies

**Figure 2:**
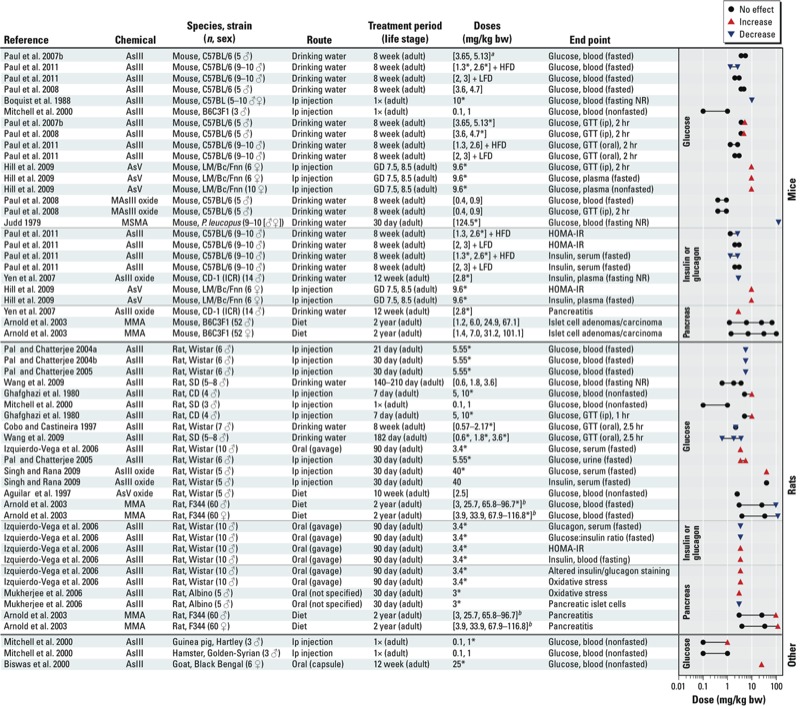
Animal studies of arsenic and end points related to glucose homeostasis. Abbreviations: AsIII, arsenite; AsIII oxide, arsenic trioxide; AsV, arsenate; AsV oxide, arsenic pentoxide; GD, gestation day; GTT, glucose tolerance test; HFD, high-fat diet; HOMA-IR, homeostasis model assessment of insulin resistance; ip, intraperitoneal; LFD, low fat diet; MAsIII oxide, methylarsine oxide; MMA, monomethylarsonate; NR, not reported. ***^a^***Bracketed information indicates that the dose was converted to mg/kg from a different dose unit presented in the publication; use of brackets can also indicate that experimental details were not explicitly stated in the paper but could be reasonably inferred. ***^b^***Notes on Arnold et al. (2003) rat findings: Effects on blood glucose in rats were only observed at 1 year of age, not at study completion at 2 years of age; the occurrence of pancreatitis was not statistically different in the high-dose group compared to controls, but there was a significant dose-related trend (*p* > 0.001) in both male and female rats. *p < 0.05; doses at which statistically significant effects were observed.

More than 20 animal studies published since 1979 were identified for this review, and they were primarily conducted with rats or mice (Figure 2). The existing studies are highly diverse, with considerable variation in the duration of treatment (1 day to 2 years), routes of administration, and in doses used in the studies. The most common routes of administration were oral, predominantly through drinking water or diet, or intraperitoneal injections. Other, less common forms of administration were gavage, oral capsules, or subcutaneous injection. Most of the studies treated animals with AsIII or arsenic trioxide, but other arsenicals have also been studied (Aguilar et al. 1997; Arnold et al. 2003; Hill et al. 2009; Paul et al. 2008). The studies also vary in experimental design and model systems used to assess end points relevant to diabetes as a health effect, ranging from urinary glucose in fasted animals (Pal and Chatterjee 2005), to blood glucose in nonfasted animals (Mitchell et al. 2000), to glucose tolerance test (Cobo and Castineira 1997; Ghafghazi et al. 1980; Hill et al. 2009; Paul et al. 2007b, 2008, 2011; Wang et al. 2009). Glucose was a commonly reported end point but findings were inconsistent across studies, which may stem from differences in the biological compartment assessed (urine, serum, plasma, whole blood) and fasting status of the animal (fasted, nonfasted, fasting status not reported) in addition to the differences in experimental design noted above related to arsenical tested, species, route of administration, and dose levels (Aguilar et al. 1997; Arnold et al. 2003; Biswas et al. 2000; Boquist et al. 1988; Ghafghazi et al. 1980; Hill et al. 2009; Izquierdo-Vega et al. 2006; Judd 1979; Mitchell et al. 2000; Pal and Chatterjee 2004a, 2004b, 2005; Paul et al. 2007b, 2008, 2011; Wang et al. 2009). Although the literature as a whole was judged inconclusive, findings from recent studies that were designed to focus more specifically on diabetes-relevant end points appear, at least qualitatively, to support a link between arsenic exposure and diabetes. Supportive findings include impaired glucose tolerance in studies with mice (Boquist et al. 1988; Hill et al. 2009; Paul et al. 2007b, 2011; Yen et al. 2007) or rats (Cobo and Castineira 1997; Ghafghazi et al. 1980; Izquierdo-Vega et al. 2006; Singh and Rana 2009; Wang et al. 2009). Measures of insulin regulation [i.e., HOMA-IR (homeostasis model assessment of insulin resistance), insulin sensitivity (Paul et al. 2011)], as well as pancreatic effects [including indicators of oxidative stress, degenerative changes in β-cells, and pancreatitis (Arnold et al. 2003; Boquist et al. 1988; Izquierdo-Vega et al. 2006; Mukherjee et al. 2006; Yen et al. 2007)], have also been reported to be affected. Results from several animal studies suggest that cotreatment with methyl donors or antioxidants (e.g., folic acid, vitamin B_12_, methionine, *N*-acetyl cysteine) may attenuate the effects of arsenic toxicity, including reductions in the degree of arsenic-induced pancreatic toxicity (Mukherjee et al. 2006) and arsenic-induced hyperglycemia (Pal and Chatterjee 2004a, 2004b, 2005). Although not directly assessing the potential diabetogenic effects of arsenic, Reichl et al. (1990) reported that cotreatment with glucose increased the survival rate in NMRI mice treated with a dose of AsIII oxide that resulted in 100% mortality when administered without the glucose (12.9 mg/kg by subcutaneous injection).

These studies suggest that animal models can be relevant to understanding the effects of arsenic on glycemic control depending on experimental design. Mice may be less susceptible than humans to arsenic toxicity, partly due to a faster metabolism and clearance of arsenic, resulting in lower internal dose of inorganic arsenic species (Paul et al. 2007b, 2008). Rats, unlike mice or humans, sequester arsenic (specifically DMA) in erythrocytes (Lu et al. 2004, 2007, 2008). It is unclear how this binding affects target organ dose of inorganic arsenic and rats are generally not recommended as a model for assessing arsenic metabolism or toxicity (NRC 1999).

## Mechanisms

**Figure 3:**
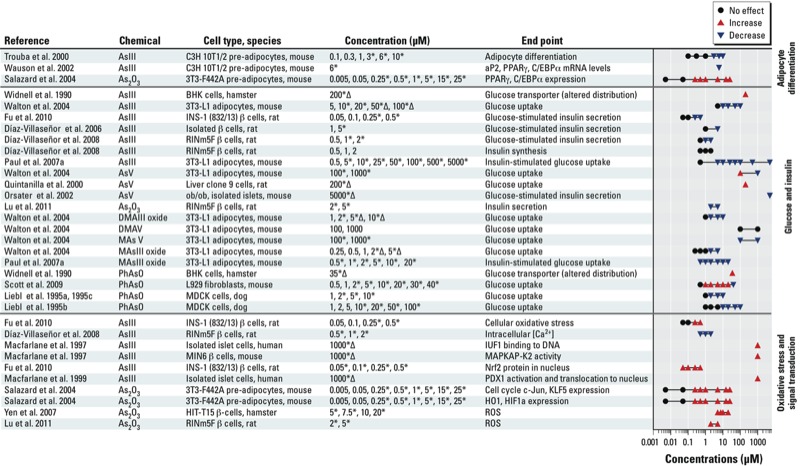
*In vitro* studies related to arsenic and diabetes. Abbreviations: Δ, cytotoxicity reported at specified concentration level; aP2, fatty acid-binding protein; As_2_O_3_, arsenic trioxide; AsIII, arsenite; AsV, arsenate; Ca, calcium; C/EBPα, CCAAT/enhancer binding protein (C/EBP alpha); DMAIII oxide, dimethylarsine oxide; DMAV, dimethylarsinate; HIF1a, hypoxia inducible factor, alpha; HO1, heme oxygenase 1; IUF1, insulin upstream factor 1 (also known as PDX1); KLF5, Kruppel-like factor 5; MAPKAP-K2, mitogen-activated protein kinase-activated protein kinase 2; MAsIII oxide, methylarsine oxide; MAsV, monosodium methylarsonate; Nrf2, transcription factor NF-E2–related factor 2; PDX1, pancreatic and duodenal homeobox 1 (also known as IUF1); PhAsO, oxophenylarsine; PPARγ, peroxisome proliferator-activated receptor γ; ROS, reactive oxygen species. *p < 0.05; doses at which statistically significant effects were observed.

A number of *in vitro* studies implicate several pathways by which arsenic can influence pancreatic β-cell function and insulin sensitivity, including oxidative stress, glucose uptake and transport, gluconeogenesis, adipocyte differentiation, and Ca^2+^ signaling (reviewed by Díaz-Villaseñor et al. 2007; Druwe and Vaillancourt 2010; Tseng 2004; see also Figure 3). Several of these pathways are discussed in more detail below, but in general the studies fall into the following categories: *a*) studies that use high concentrations of arsenic (≥ 1 mM) to examine stress response in various cell types, although the concentrations used limit interpretation because they are not considered physiologically relevant, resulting in cytotoxicity; *b*) studies that test lower concentrations (< 100 μM) of arsenic and report inhibition of insulin signaling and insulin-dependent glucose uptake by adipocytes or myotubes (Paul et al. 2007b; Walton et al. 2004; Yen et al. 2010); and *c*) studies in insulinoma cell lines or isolated pancreatic islets that suggest that the mechanisms by which arsenic affects β-cells to inhibit insulin expression and/or secretion are concentration dependent (Díaz-Villaseñor et al. 2006, 2008; Fu et al. 2010; Pi et al. 2007). At relatively low concentrations (in the submicromolar range) certain adaptive cellular responses to arsenic-induced oxidative stress [i.e., induction of antioxidant enzymes and reduced reactive oxygen species (ROS)] may result in an impairment of glucose-stimulated insulin secretion (Fu et al. 2010; Pi et al. 2007). High concentrations result in irreversible damage (including oxidative damage) to β-cells followed by apoptosis or necrosis (Macfarlane et al. 1997, 1999; Ortsater et al. 2002).

*Influence of inorganic arsenic on glucose-stimulated insulin secretion in pancreatic* β*-cells.* Chronic oxidative stress leading to oxidative damage has long been implicated in β-cell dysfunction in diabetes. Oxidative stress is also implicated in many aspects of arsenic toxicity, and a recent *in vitro* study suggests that transcription factor NF-E2–related factor 2 (Nrf2)-mediated antioxidant response may influence arsenite-induced impairment of glucose-stimulated insulin secretion in β-cells at low concentrations of arsenite (Fu et al. 2010). The transcription factor Nrf2 is a key cellular component that defends cells against toxicities of oxidants and electrophiles by regulating both constitutive and inducible expression of many antioxidant/detoxification enzymes (Fu et al. 2010; He and Ma 2010). Although antioxidants are generally considered protective for cells, this same Nrf2-driven induction of endogenous antioxidant enzymes meant to maintain intracellular redox homeostasis and limit oxidative damage may also have a negative impact on insulin secretion by diminishing the availability of ROS, such as hydrogen peroxide (H_2_O_2_). Reactive oxygen species’ signals produced during glucose metabolism are becoming recognized as intracellular regulators of glucose-stimulated insulin secretion acting to increase insulin secretion (Leloup et al. 2009; Pi et al. 2007, 2010).

Thus, the Nrf2-mediated antioxidant response appears to play paradoxical roles in β-cell function by *a*) blunting glucose-triggered ROS signaling and thus resulting in reduced glucose-stimulated insulin secretion, and *b*) protecting β-cells from oxidative damage and subsequent apoptosis/necrosis (Fu et al. 2010). Chronic exposure to inorganic arsenic and the production of its methylated trivalent metabolites have been linked to oxidative stress; however, at the levels generally expected in low-to-moderate human exposures, they are not likely to reach cytotoxic concentrations sufficient to cause irreversible damage, although at these levels they may activate Nrf2. Therefore, premise *a* above is potentially more relevant to β-cell dysfunction in the context of low-level environmental arsenic exposure, whereas premise *b* might be associated with β-cell damage and failure induced by high doses of arsenic.

*Influence of trivalent arsenicals on glucose uptake in adipocytes and skeletal muscle cells.* Type 2 diabetes is characterized by disruptions in whole-body glucose homeostasis due to insulin resistance and impaired glucose utilization by peripheral tissues, including skeletal muscle and adipose tissue. Results of tissue culture studies suggest that arsenite and/or its methylated trivalent metabolites cause insulin resistance in adipocytes by inhibiting insulin signaling and insulin-activated glucose uptake. Arsenite can also interfere with the formation of insulin-sensitive adipocytes and myotubes by inhibiting adipogenic and myogenic differentiation (Salazard et al. 2004; Trouba et al. 2000; Walton et al. 2004; Yen et al. 2010).

Arsenite and its metabolites interact with a number of elements involved in insulin signaling, including insulin receptor substrate (IRS), phosphatidylinositol-3 kinase (PI3K), AKT, phosphoinositide-dependent kinase (PDK), and protein kinase C (PKC). AKT belongs to a class of enzymes important in regulating glucose metabolism, cell proliferation, apoptosis, transcription, and cell migration (Paul et al. 2007a; Walton et al. 2004). Insulin stimulates glucose uptake by binding to the insulin receptor and activating the IRS-1, IRS-2, PI3K, PDK, AKT, and/or PKC-ζ/PKC-λ signaling pathway(s) (Choi and Kim 2010; Standaert et al. 1999). Activation of PKC-ζ and PKC-λ stimulates Ras-related protein (RAB4A) activity, the association of RAB4A with kinesin-like protein KIF3B, and the interaction of KIF3B with microtubules. This process is essential for recruitment of glucose transporter type 4 (GLUT4) to the cytoplasmatic membrane and for insulin-dependent glucose uptake (Imamura et al. 2003; Lee et al. 2010). Subcytotoxic concentrations in the micromolar range of arsenite and its methylated trivalent metabolites, MMAIII and DMAIII, inhibit insulin-stimulated glucose uptake in cultured adipocytes by interfering with the phosphorylation of AKT-dependent mobilization of GLUT4. Arsenite and MMAIII inhibit PDK-catalyzed phosphorylation of AKT in the insulin signaling cascade; DMAIII inhibits GLUT4 translocation by interfering with the signaling step(s) downstream from AKT (Paul et al. 2007a; Walton et al. 2004). The adaptive antioxidant response associated with prolonged exposure to relatively low concentrations of arsenite in the 1–2 µM range have also been associated with suppression of insulin-stimulated AKT phosphorylation and glucose uptake in 3T3-L1 adipocytes causing an insulin resistant phenotype (Xue et al. 2011).

Insulin resistance is a hallmark of diabetes and the role of adipocytes in mediating insulin resistance is an active area of research. A number of studies have assessed the impact of arsenic on adipocytes. Arsenite inhibits and reverses differentiation of adipocytes by disrupting the expression of the genes involved in adipogenesis (Wauson et al. 2002). Expression of both peroxisome proliferator-activated receptor-γ (PPARγ) and CCAAT/enhancer-binding protein α (C/EBPα) is required for phenotypic differentiation of adipocytes, and arsenite inhibits expression of both of these transcription factors. Arsenite disrupts the interaction between PPARγ and its coactivator retinoid X receptor alpha (RXRα). Arsenic trioxide also inhibits AKT binding to PPARγ (Wang et al. 2005). Inhibition of these transcription factors reduces expression of PPARγ and C/EBPα target genes: adipocyte fatty acid binding protein (A-FABP), which is involved in preadipocyte differentiation, and p21, a protein whose expression is tightly regulated during adipogenesis (Wang et al. 2005; Wauson et al. 2002). Inhibition of p21 leads to activation of preadipocyte proliferation, thereby inhibiting adipocyte differentiation (Wang et al. 2005).

Myogenesis is associated with the development of the insulin-responsive glucose transport system and there are indications that arsenite may have similar effects on myogenic differentiation; however, this has not been studied to the same extent as its effects on adipocytes. Pathways mediating muscle differentiation include insulin-dependent activation of AKT/mTOR/p70 S6 kinase1/MEF2C/MYOD/MYOG signaling (Conejo et al. 2002; Xu and Wu 2000). Low concentrations (e.g, 20 nM) of arsenite have been shown to delay the differentiation of muscle cells from myoblasts to myotubes by repressing the transcription factor myogenin (Steffens et al. 2010). Arsenite also significantly decreases the phosphorylation of AKT and its downstream targets, mTOR and p70 S6 kinase1 proteins, during myogenic differentiation (Yen et al. 2010). Inhibition of AKT by arsenite was also demonstrated in muscle cells (Yen et al. 2010), and may lead to a reduction in glucose uptake in this tissue (Díaz-Villaseñor et al. 2007).

## Conclusions and Research Needs

Overall, data from human studies included in this review support an association between inorganic arsenic and diabetes in populations with arsenic drinking-water levels of > 500 µg/L (Lai et al. 1994; Nabi et al. 2005; Rahman et al. 1998, 1999; Tsai et al. 1999; Tseng et al. 2000b; Wang et al. 2003), but the currently available evidence was considered insufficient to conclude that arsenic is associated with diabetes in individuals with low-to-moderate exposure (< 150 µg/L in drinking water). Stronger evidence of associations at lower levels of exposure based on some recent studies with better measures of outcome and exposure support the need for further research in populations with low-to-moderate exposure levels. Weaknesses noted in the epidemiological literature review included a lack of prospective studies, use of death certificates or self-reported diagnosis for ascertainment of diabetes, and ecological methods of exposure assessment. Because of these limitations, the evidence of effects at high arsenic exposure levels ranged from limited to sufficient, but did not reach the threshold for a sufficient classification.

Research needs identified as a result of this literature review are summarized in Table 3. Prospective studies in areas of lower exposure (e.g., parts of North America other than arsenic-endemic regions) with individual measurements of exposure prior to disease incidence are needed. However, the utilization of existing cohorts (such as the Strong Health Study), nested case–control designs, and follow-up of cross-sectional populations such as NHANES is also recommended. Additional consideration of the results from the recent HEALS study in Bangladesh (Chen et al. 2010), which do not align with findings from other studies in areas of moderate-to-high exposure, would also be helpful to better understand factors that influence the generalizability of associations reported based on other study populations. Research on interactions between arsenic exposure and factors such as body mass index (BMI), diet, levels of physical activity, co-exposures including metals that occur with arsenic, duration of exposure, and timing of exposure (i.e., the importance of early life or prenatal exposures) may help address this issue. In addition, future studies should include consideration of gene × environment interactions, including studies of polymorphisms in genes related to arsenic metabolism and diabetes susceptibility.

**Table 3:** Research needs.

Epidemiology
Prospective studies with incident cases for diabetes, especially at lower exposure ranges
Consider utilizing existing cohorts, nested case–control design, and follow-up of cross-sectional populations
Impact of early-life exposures
Impact of arsenic metabolism
Impact of diet, BMI, and physical activity
Genetic susceptibility related to both response to arsenic and diabetes
Epigenetic research related to mechanisms
Investigate potential increased risk for type 1 diabetes and gestational diabetes
Exposure
Exposure data on other arsenicals, i.e., thioarsenicals, roxarsone
Method development for urinary DMAIII and MMAIII and measurement of arsenic metabolites in blood
Co-exposure between arsenic and other chemicals including metals
Cost-effective strategies for analysis and markers of seafood arsenic
Better characterization of other biomarkers of exposure [i.e., toe- and fingernails (noninvasive and reflect long-term exposure), saliva, buccal cells, target tissues]
Validate spot urine findings with 24-hr urine samples for a sample of the study population
Animal and in vitro
Identify animal models appropriate for arsenic-induced diabetes
Need to consider internal dose
Epigenetic research that includes an emphasis on developmental effects
Assess low-concentration effects in vitro
Mechanisms of glucose homeostasis in other tissues (in vitro)

Given its well-established role as a risk factor for diabetes, the impact of obesity as a potential modifying factor needs to be better addressed, especially in countries such as the United States and Mexico where overweight and obesity are epidemic (WHO 2012). Average BMI in Bangladesh and Taiwan, where the association between arsenic exposure and diabetes was stronger, is much lower than in the United States and Mexico. For example, approximately 80% of study participants in the HEALS study in Bangladesh had a BMI of < 22 (Chen et al. 2010) whereas 68% of study participants included in the analysis of NHANES 2008 had a BMI of ≥ 25 (Navas-Acien et al. 2008). In the Mexico studies, 34–50% of participants had a BMI of > 30 (Coronado-González et al. 2007; Del Razo et al. 2011). Information on BMI was not presented in most of the studies conducted in Taiwan except for Tseng et al. (2000b), where the average BMI was 24.5 kg/m^2^; although as a population, the prevalence of overweight/obesity is higher in Taiwan compared to Bangladesh and lower compared to the United States (Huang 2008; WHO 2012). Many of the recent studies considered BMI as a potential confounding factor (Chen et al. 2010; Coronado-González et al. 2007; Del Razo et al. 2011; Ettinger et al. 2009; Kim and Lee 2011; Lai et al. 1994; Navas-Acien et al. 2008, 2009a; Rahman et al. 1999; Steinmaus et al. 2009a, 2009b; Tseng et al. 2000b), but the issue of obesity as an effect modifier or potential intermediate on a causal pathway between arsenic and diabetes has not been well-explored in the existing literature.

The experimental animal literature as a whole was judged inconclusive, but findings from recent studies that focus on diabetes-relevant end points appear consistent with those human studies that support a link between arsenic exposure and diabetes. Moreover, the animal studies implicate several pathways by which arsenic may influence pancreatic β-cell function and insulin sensitivity and suggest novel biomarkers for understanding pathways of response to arsenic in human populations. However, animal studies need to be designed to be relevant to human exposures in terms of internal dose. Use of specific inbred strains susceptible to diabetes and metabolic syndrome may also be informative. Application of systems toxicology approaches within the framework utilized by the NIEHS and others in studying relevance of the “toxome” [a comprehensive list of all pathways of toxicity (Hartung and McBride 2011)] to the “diabetome” [a conceptual platform placing a disease, diabetes, onto a network perspective and linking diabetes phenotypic features to all known diabetes-related genes (modified from Goh and Choi 2012)] may be innovative and stimulate new information on key signaling pathways that connect arsenic to diabetes.

Overall, animal studies need to be designed to specifically evaluate the influence of arsenic on the development of diabetes, using modern methods and well characterized end points for diabetes. Blood glucose levels, both fasting and fed, as well as insulin levels were identified as appropriate end points for animal studies. The influence of adiposity on the development of arsenic-induced diabetes could be explored more fully in animal models by quantitating fat mass and distribution in both white and brown adipose tissues.

Improved methodologies are needed for more accurate environmental exposure assessments as well as for internal dosemetrics and biologically based measurements that integrate all and differentiate among exposures, metabolites, and toxicities. Some of the newer proposed biomarkers (e.g., toe- and fingernails, saliva, buccal cells) need to be further characterized in terms of their relationships to external exposures and validated.

## Correction

There were errors in the manuscript originally published online. In the “Conclusion” of the Abstract and in the titles of Tables 1 and 2, the exposure levels for arsenic should have been given as “µg/L” instead of “µg/µL” or “ppm.” The errors have been corrected here.

## Supplemental Material

(184 KB) PDF
