# Supplementary material for: Evaluation of the Association between Arsenic and Diabetes: A National Toxicology Program Workshop Review
Source: Environ Health Perspect. 2012 Aug 10;120(12):1658–70. doi: 10.1289/ehp.1104579 (PMC3548281; doi:10.1289/ehp.1104579)
Supplement: (184 KB) PDF [file ehp.1104579.s001.pdf]

SUPPLEMENTAL MATERIAL

EVALUATION OF THE ASSOCIATION BETWEEN ARSENIC AND DIABETES: A NATIONAL TOXICOLOGY PROGRAM WORKSHOP REVIEW

Elizabeth A. Maull, Habibul Ahsan, Joshua Edwards, Matthew P. Longnecker, Ana Navas-Acien, Jingbo Pi, Ellen K. Silbergeld, Miroslav Styblo, Chin-Hsiao Tseng, Kristina A. Thayer, Dana Loomis

TABLE OF CONTENTS

Literature search.. 2

Supplemental Material Figure S1. Study Flow Information.. 3

Supplemental Material, Table S1: Summary of Occupational Studies.. 4

Expanded discussion on "Accounting for Arsenic of Seafood Origin"... 5

Supplemental Material, Table S2: Common Forms of Arsenic.. 1

References Cited in Supplemental Materials... 12

Literature search

MeSH-based PubMed search: "Arsenic"[Mesh] AND (("obesity"[mh] OR "body mass index"[mh] OR "weight gain"[mh] OR "adipogenesis"[mh] OR "adipose tissue"[mh] OR "adipokines"[mh] OR "adiponectin"[mh] OR "leptin"[mh] OR "resistin"[mh]) OR ("diabetes mellitus"[mh] OR "glucose metabolism disorders"[mh] OR "insulin"[mh] OR "insulin resistance"[mh] OR "blood glucose"[mh] OR "islets of langerhans"[mh])) AND (("obesity"[mh] OR "body mass index"[mh] OR "weight gain"[mh] OR "adipogenesis"[mh] OR "adipose tissue"[mh] OR "adipokines"[mh] OR "adiponectin"[mh] OR "leptin"[mh] OR "resistin"[mh]) OR ("diabetes mellitus"[mh] OR "glucose metabolism disorders"[mh] OR "insulin"[mh] OR "insulin resistance"[mh] OR "blood glucose"[mh] OR "islets of langerhans"[mh]))

Keyword-strategy to search "new" un-indexed articles: Arsenic AND ((diabetes OR "glucose tolerance" OR "glucose intolerance" OR hyperglycemia OR hypoglycemia OR insulin OR "blood glucose" OR "metabolic syndrome" OR "syndrome x" OR "islets of langerhans") OR (obes* OR "body mass index" OR "body fat" OR "weight gain" OR adipos* OR adipogen* OR adipokine* OR leptin OR resistin OR adiponectin*)) AND (publisher[sb] OR "in process"[sb])

Supplemental Material Figure S1. Study Flow Information

References identified from PubMed search run through 12/15/2010 (n = 108)

↓

Excluded (n = 70)

- No original data (review/commentary/letter; n = 10)
- No relevant outcome reported in study (n=51) or supportive material (n = 8)
- Non-English (n = 1)

Included from PubMed Search (n = 38)

+

Included from reviewing the reference lists in the primary literature or review articles (n = 38)*

<u>Total included (n=76)</u>**

Studies with human data (n = 36)

Studies with animal data (n = 21)

Studies with *in vitro* or *ex vivo* data (n = 21)

* Two studies were not yet published at the time of the workshop but one or more members of the panel provided information about them during the breakout group discussions; both studies have been published subsequent to the workshop (Del Razo et al. 2011; Paul et al. 2011).

** Two studies contained data for more than one evidence stream, i.e., human/animal (Wang et al. 2009) and animal/*in vitro* (Yen et al. 2007)

Supplemental Material, Table S1: Summary of Occupational Studies

Refrence and Study Design	Subjects	Diabetes Diagnosis	Main Finding[a]	Exposure Comparison	Factors Considered in Analysis
**(Bartoli et al. 1998)** Retrospective	Italy (Tuscany) art glass workers, n=3,390 ♂, 488 deaths	death certificate	0.34 (95% CI: 0.09, 0.88) SMR	workers vs Tuscany	age, gender, calendar year
**(Mabuchi et al. 1980)[b]** Retrospective	US (Baltimore, MD) pesticide workers, ♂♀, 240 deaths	death certificate	0.47 (95% CI: 0.12, 1.88) SMR	workers vs Baltimore	age, sex, period
**(Lubin et al. 2000)** Retrospective	US (MT) Lee-Fraumeni, smelter, n=8,014 ♂, 4,912 deaths	death certificate	0.83 (95% CI: 0.63, 1.08) SMR	workers vs US	age
**(Enterline and Marsh 1982)[b]** Retrospective	US (WA) smelter workers, n=2,802 ♂, 1,061 deaths	death certificate	1 (95% CI: 0.78, 1.28) adjOR	yes (referent) vs unexposed	age
**(Rahman et al. 1996)** case-control	Sweden (southeastern) art glass workers, ♂, 2,333 deaths	death certificate	1.4 (95% CI: 0.9, 2.1) MH-OR	likely exposed vs unexposed	age
**(Rahman and Axelson 1995)** case-control	Sweden (St. Orjan) smelter workers, n=369 ♂, 43 deaths	death certificate, clinical support	3.3 (95% CI: 0.5, 30) MH-OR	"total exposed" vs no exposure	age and sex
**(Jensen and Hansen 1998)[c]** cross-sectional	Denmark (NR) exposed workers, n=64 ♂♀	HbA1c >7%	4.43 (95% CI: 0.47, 42) RR	exposed workers vs reference group	age
**(Lagerkvist and Zetterlund 1994)[c]** cross-sectional	Sweden (Northern) smelter workers, n=89 ♂	self-report	9.61 (95% CI: 0.53, 173) RR	smelter vs car factory workers	unadjusted

[a]Identification of main findings was based on the following strategy: For studies that did not report a significant association between arsenic exposure and a health outcome at any exposure level, the main summary finding was based on the highest exposure group compared to the referent group (e.g., 4th quartile versus 1st quartile). When a study reported a significant association between arsenic exposure and a health outcome the main finding was based on lowest exposure group where a statistically significant association was observed (e.g., 3rd quartile versus 1st quartile).
[b]Calculated by entering data presented in publication into OpenEpi software (Dean et al. 2011).
[c]Relative risk and 95% confidence interval as estimated by Navas-Acien et al. (2006).

Expanded discussion on “Accounting for Arsenic of Seafood Origin”

Most human biomonitoring studies report levels of total arsenic which includes inorganic (i.e., arsenite, arsenate) and organic arsenic compounds (mainly arsenobetaine, arsenosugars, and arsenolipids) and their metabolites. It is important to discern how much of the total arsenic measurement is due to intake of inorganic arsenic because organic arsenicals, mostly found in seafood, are generally considered to be of little toxicological significance. In many cases only total urinary levels of arsenic are reported in human biomonitoring studies and it can be challenging to reach conclusions on associations between inorganic arsenic and diabetes or other health measures. This is less of a challenge when study participants are exposed to higher levels of arsenic from drinking water, occupation, or proximity to an industrial or mining site with arsenic contamination. In these cases, urinary arsenic is generally assumed to be mostly from exposure to inorganic arsenic and other exposure to pollutants is likely. However, in studies of the general population like NHANES it is more difficult to identify the portion of urinary arsenic that can be attributed to intake of organic arsenic, mostly due to seafood consumption (Longnecker 2009; Navas-Acien et al. 2009; Steinmaus et al. 2009).

NHANES includes measurement of total arsenic and seven arsenic species - four inorganic-related forms [arsenite, arsenate, the methylated metabolites produced in the body dimethylarsinic acid (DMA), and monomethylarsonic acid (MA)] and three organic forms (arsenobetaine, arsenocholine, and trimethylarsine oxide). The measurement of total arsenic is a separate chemical analysis and reflects other arsenicals in addition to the seven species measured, i.e., total arsenic is not the sum of the seven specific species. With respect to the species measured in NHANES it is important to take into account some issues. First, the species more readily reflecting inorganic arsenic exposure (arsenite, arsenate, and MA) are undetectable in the majority of the general population and cannot be used in epidemiologic studies. Second, although DMA is the major metabolite of inorganic arsenic is also a metabolite of arsenosugars and arsenolipids and therefore reflects both exposures to inorganic and organic forms of arsenic. Third, arsenocholine and trimethylarsine oxide are not the major forms of arsenic found in seafood and are not considered to be significant sources of exposure to organic arsenic. This is supported by the finding that these forms of arsenic were only detected in a small number of

urine samples in NHANES, arsenocholine (1.8%) and trimethylarsine oxide (0.3%). Although it is worth noting that arsenobetaine is the predominant urinary metabolite of arsenocholine, at least in rats, mice and rabbits (Marafante et al. 1984). In any event, urinary arsenobetaine due to the ingestion of arsenocholine would still ultimately be attributed to consumption of organic arsenic. Urine can also contain thioarsenicals which were not analyzed in this study.

Three general approaches have been proposed to account for organic arsenic of seafood origin in NHANES (1) restrict the analysis to participants not likely to have consumed seafood close to the time of sample collection by restricting the sample to participants with very low or undetectable arsenobetaine (Navas-Acien et al. 2008, 2009), (2) statistically adjust for urinary levels of arsenobetaine or blood mercury as markers for seafood consumption (Navas-Acien et al. 2008, 2009), and (3) subtract from the total urinary arsenic measurement any organic arsenicals that were detected in NHANES participants, i.e., arsenobetaine and arsenocholine (if above the detection limit)[1](Steinmaus et al. 2009). These three general strategies lead to different conclusions on the association between inorganic arsenic and diabetes in NHANES. In the initial 2008 publication by Navas-Acien et al., the authors controlled for seafood intake by restricting the analysis to participants who did not report seafood intake in the 24-hour period prior to sample collection and adjusting total urine arsenic for objective measures of seafood intake (urinary arsenobetaine and blood mercury). The result after correcting for other factors (e.g., age, sex, body mass index, etc.) was a 3.58-fold increase (95% CI, 1.18-10.83) in diabetes at the 80th (16.5 µg/L) versus the 20th (3.0 µg/L) percentiles of total urinary arsenic. Using the same NHANES 2003-2004 data as Navas-Acien et al. (2008), but taking the approach of subtracting arsenobetaine and arsenocholine from total arsenic, Steinmaus et al.(2009) found no association between arsenic and diabetes when comparing participants ≥ 80th vs. ≤ 20th percentiles of total urinary arsenic, OR 0.88 (95% CI 0.39-1.97). One criticism of the approach used by Steinmaus et al.(2009) is that subtracting arsenobetaine and arsenocholine from the total will not remove other organic forms of arsenic not specifically measured in NHANES but included in the measure of total urinary arsenic) (Navas-Acien et al. 2009). In addition, DMA is the main metabolite of arsenosugars and arsenolipids and the approach used by Steinmaus et al. (2009)

[1]Steinmaus et al. Steinmaus C, Yuan Y, Liaw J, Smith AH. 2009. Low-level population exposure to inorganic arsenic in the United States and diabetes mellitus: A reanalysis. Epidemiology 20(6): 807-815. Did not consider trimethylarsine oxide because it was only detected in 0.3% NHANES participants.

would not account for DMA that may be of seafood origin. In addition, the portion of DMA in NHANES due to the metabolism of inorganic arsenic or arsenosugars/arsenolipids from seafood likely differs at lower levels of total urinary arsenic compared to higher levels. In NHANES 2003–2004, DMA was the major contributor to total urinary arsenic at lower levels (<20 µg/L), with a median contribution of 53.8%. At higher levels of total urinary arsenic (≥ 50 µg/L) arsenobetaine was the major form with a median contribution of 62.7% to the total (Caldwell et al. 2009). In participants where arsenobetaine is the major contributor to total urinary arsenic, DMA in urine was also likely to reflect exposure to organic arsenicals in seafood, since DMA is a metabolite of arsenosugars and arsenolipids that co-occur with arsenobetaine in seafood. In NHANES 2003-2004, the correlation coefficient between arsenobetaine and DMA was 0.48 (Navas-Acien A, personal communication). These complexities can be avoided by controlling for seafood ingestion although there may be a loss of statistical power because the number of NHANES participants included in the analysis will be reduced.

Navas-Acien et al. (2009) extended the original NHANES 2003-2004 analysis to include data from NHANES 2003-2006 but this time accounted for organic arsenic of seafood origin by restricting the analysis to participants with undetectable arsenobetaine (≤ 0.4 µg/L; n = 381, 62 with diabetes). After adjustment for sociodemographic and diabetes risk factors, the OR for diabetes was 2.60 (95% CI: 1.12– 6.03) comparing participants at the 80th versus the 20th percentiles of total urine arsenic (7.4 vs. 1.6 µg/L) and 4.26 (95% CI: 0.83-21.8) in participants ≥ 80th vs. ≤ 20th percentile of total urine arsenic. The impact of inadequately accounting for seafood-derived arsenicals appears to be more of a factor at higher total urinary arsenic levels where organic arsenic from seafood (arsenobetaine) is the predominant contributor as noted above (Caldwell et al. 2009). Navas-Acien et al. (2009) found considerable potential for exposure misclassification when inorganic arsenic was estimated by total urine arsenic minus arsenobetaine and arsenocholine compared to using total urine arsenic in participants with undetectable arsenobetaine. Total arsenic minus arsenobetaine and arsenocholine at the 20th, 50th, 80th, 90th, and 99th percentiles was 2.7, 11.9, 18.4, and 73.6 µg/L, respectively, while the distribution of total urine arsenic among participants with undetectable urine arsenobetaine at the same percentiles was 1.9, 3.9, 7.7, 11.8, and 30.4 µg/L, respectively.

Supplemental Material, Table S2: Common Forms of Arsenic

Common Name[a] (Systematic Name)	CAS No.	Abbreviation & Synonyms	Formula	Structure	Description
**INORGANIC**					
Arsenic	7440-38-2	As	As		
Arsenate	12523-21-6;<br>7778-43-0 (sodium salt)	As(V)	AsO_4^{3-}<br>(in basic conditions)	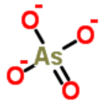	trace to low levels in most foods; a major form in water; considered highly toxic and carcinogenic
Arsenic acid	7778-39-4		H_3AsO_4<br>(in acidic conditions)	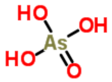	
Arsenite	7784-46-5<br>(sodium salt)	As(III)	AsO_3^{3-}<br>(in basic conditions)	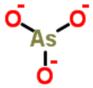	trace to low levels in most foods; considered highly toxic and carcinogenic
Arsenous acid	13464-58-9		H_3AsO_3<br>(in acidic conditions)	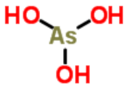	
Arsenic trioxide	1327-53-3	As(III) oxide, As trioxide,<br>white As, arsenolite	As_2O_3	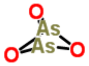	commercial compound of arsenic used in the manufacturing other arsenic compounds used as wood preservatives, insecticides, and herbicides; also used in metallurgical processes, manufacturing of glass and ceramics, and as an anticancer drug; can be found in nature but is more commonly associated with smelting

Supplemental Material, Table S2: Common Forms of Arsenic (cont.)

Common Name[a] (Systematic Name)	CAS No.	Abbreviation & Synonyms	Formula	Structure	Description
**ORGANIC**					
Arsenobetaine	64436-13-1	AB or AsB, “fish arsenic”	$C_5H_{11}AsO_2^-$	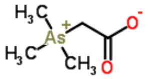	major organic arsenic species in most seafoods; generally considered non-toxic
Arsenosugars[b]				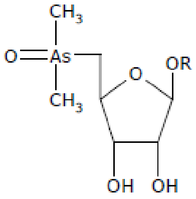	major (edible algae) or significant (mollusks) arsenic species in many seafoods; mostly metabolized to DMA in humans
Arsenolipids[c]				e.g. 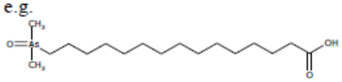	newly discovered arsenic species present in fish oils and fatty fish; likely to be present in other seafoods as well; mostly metabolized to DMA
Arsenocholine	39895-81-3	AC or AsC	C5H14AsO	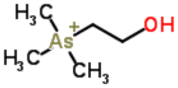	trace organic arsenic species found in seafood; readily oxidized to arsenobetaine in biological systems
Arsenocholine	39895-81-3	AC or AsC	$C_5H_{14}AsO$	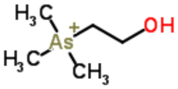	trace organic arsenic species found in seafood; readily oxidized to arsenobetaine in biological systems
Trimethylarsine oxide	4964-14-1	TMAO	C_3H_9AsO	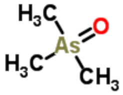	minor organic arsenic species found in seafood; major product of As metabolism in some bacterial and animal species

Supplemental Material, Table S2: Common Forms of Arsenic (cont.)

Common Name[a] (Systematic Name)	CAS No.	Abbreviation & Synonyms	Formula	Structure	Description
**ORGANIC**					
Dimethylarsinate		DMA	$C_2H_6AsO_2^-$	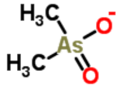	minor arsenic species in seafoods and some terrestrial foods; the major human urine metabolite of iAs, arsenosugars, and arsenolipids
Dimethylarsinic acid	75-60-5		$C_2H_7AsO_2$	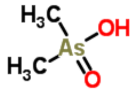	
Dimethylarsinite		DMA(III)	$C_2H_6AsO^-$	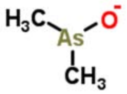	not detected in foods; detected in some human urine samples as a metabolite of iAs; a very unstable (reactive) species that is very difficult to measure; highly toxic species considered by some researchers to be central to arsenic's mode of toxic action
Dimethylarsinous acid	55094-22-9		C_2H_7AsO	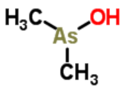	
Methylarsonate	51952-65-9	MA, MMA, monomethylarsonate	$CH_3AsO_3^{2-}$	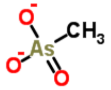	trace arsenic species of some seafoods and terrestrial foods; a significant human urine metabolite of iAs
Methylarsonic acid, monomethylarsonic acid	124-58-3		CH_5AsO_3	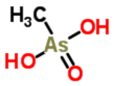	
Methylarsonite		MA(III), MMA(III), monomethylarsonite	$CH_3AsO_2^{2-}$	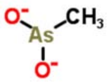	not usually detected in foods; detected in some human urine samples as a metabolite of iAs; a toxic species thought to be important for arsenic's mode of toxic action
Methylarsonous acid, monomethylarsonous acid	25400-23-1		CH_5AsO_2	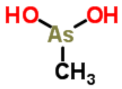	

Supplemental Material, Table S2: Common Forms of Arsenic (cont.)

Common Name (Systematic Name)	CAS No.	Abbreviation & Synonyms	Formula	Structure	Description
**ORGANIC**					
Roxarsone	121-19-7		$C_6H_6AsNO_6$	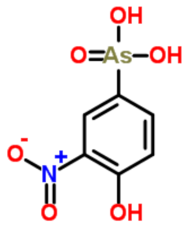	approved by the FDA for use as a medicinal feed additive; used in animal and poultry feeds as antimicrobials; reported to be excreted unchanged in manure. Additional research is needed to confirm

From International Programme on Chemical Safety (IPCS) (2001), ATSCR (2007), Caldwell et al.(2009), European Food Safety Authority (EFSA) (2009), ChemSpider(2010)

(a) Table is incomplete due to a lack of complete information about all chemical species of arsenic in food and about the metabolites generated from these chemicals forms.
(b) Over 20 arsenosugars have been reported as natural products; they differ by having different R groups on the aglycone portion of the molecule, and by replacing the oxygen on the arsenic atom with either a sulfur atom or a third methyl group (see Francesconi and Edmonds (1997)). Most of the arsenic present as arsenosugars, however, is contained in just four compounds based on the structure drawn above and with (i) R=CH2CHOHCH2OH (European Food Safety Authority 2009).
(c) Nine arsenolipids have been reported so far as natural products, all of which contain the dimethylarsinoyl group [(CH3)2As(0)-] bound to either one of several long chain fatty acids, or to long chain hydrocarbons. Many more arsenolipids are present in foods – their structures are currently unknown (European Food Safety Authority 2009).

References Cited in Supplemental Materials

Agency for Toxic Substances and Disease Registry (ATSDR). 2007. Toxicological profile for Arsenic. Atlanta, GA: U.S. Department of Health and Human Services, Public Health Service. http://www.atsdr.cdc.gov/toxpro2.html [accessed 12 December 2011].

Bartoli D, Battista G, De Santis M, Iaia TE, Orsi D, Tarchi M, et al. 1998. Cohort study of art glass workers in Tuscany, Italy: mortality from non-malignant diseases. Occup Med (Lond) 48(7): 441-445.

Caldwell KL, Jones RL, Verdon CP, Jarrett JM, Caudill SP, Osterloh JD. 2009. Levels of urinary total and speciated arsenic in the US population: National Health and Nutrition Examination Survey 2003-2004. J Expo Sci Environ Epidemiol 19(1): 59-68.

ChemSpider. 2010. http://www.chemspider.com [accessed 12 December 2011].

Dean A, Sullivan K, Soe M. 2011. OpenEpi: Open Source Epidemiologic Statistics for Public Health, Version 2.3.1. www.OpenEpi.com, updated 2011/23/06 [accessed 8 December 2011].

Enterline PE, Marsh GM. 1982. Cancer among workers exposed to arsenic and other substances in a copper smelter. Am J Epidemiol 116(6): 895-911.

European Food Safety Authority. 2009. EFSA Panel on Contaminants in the Food Chain (CONTAM); Scientific Opinion on Arsenic in Food.EFSA Journal 2009; 7(10):1351. [198 pp.]. doi:10.2903/j.efsa.2009.1351. http://www.efsa.europa.eu/en/scdocs/scdoc/1351.htm [accessed 12 December 2011].

Francesconi KA, Edmonds JS, Sykes AG. 1997. Arsenic and Marine Organisms. Advances in Inorganic Chemistry Volume 44: 147-189.

IPCS. 2001. Arsenic and arsenic compounds (2nd edition). Environmental Health Criteria 224. http://www.who.int/ipcs/publications/ehc/ehc_alphabetical/en/index.html [accessed 12 December 2011].

Jensen GE, Hansen ML. 1998. Occupational arsenic exposure and glycosylated haemoglobin. Analyst 123(1): 77-80.

Lagerkvist BJ, Zetterlund B. 1994. Assessment of exposure to arsenic among smelter workers: a five-year follow-up. Am J Ind Med 25(4): 477-488.

Longnecker MP. 2009. On confounded fishy results regarding arsenic and diabetes. Epidemiology 20(6): 821-823; discussion e821-822.

Lubin JH, Pottern LM, Stone BJ, Fraumeni JF, Jr. 2000. Respiratory cancer in a cohort of copper smelter workers: results from more than 50 years of follow-up. Am J Epidemiol 151(6): 554-565.

Mabuchi K, Lilienfeld AM, Snell LM. 1980. Cancer and occupational exposure to arsenic: a study of pesticide workers. Preventive Medicine 9(1): 51-77.

Marafante E, Vahter M, Dencker L. 1984. Metabolism of arsenocholine in mice, rats and rabbits. Sci Total Environ 34(3): 223-240.

Navas-Acien A, Silbergeld EK, Pastor-Barriuso R, Guallar E. 2008. Arsenic exposure and prevalence of type 2 diabetes in US adults. JAMA 300(7): 814-822.

Navas-Acien A, Silbergeld EK, Pastor-Barriuso R, Guallar E. 2009. Rejoinder: Arsenic exposure and prevalence of type 2 diabetes: updated findings from the National Health Nutrition and Examination Survey, 2003-2006. Epidemiology 20(6): 816-820; Discussion e811-812.

Navas-Acien A, Silbergeld EK, Streeter RA, Clark JM, Burke TA, Guallar E. 2006. Arsenic exposure and type 2 diabetes: a systematic review of the experimental and epidemiological evidence. Environ Health Perspect 114(5): 641-648.

Rahman M, Axelson O. 1995. Diabetes mellitus and arsenic exposure: a second look at case-control data from a Swedish copper smelter. Occup Environ Med 52(11): 773-774.

Rahman M, Wingren G, Axelson O. 1996. Diabetes mellitus among Swedish art glass workers--an effect of arsenic exposure? Scand J Work Environ Health 22(2): 146-149.

Steinmaus C, Yuan Y, Liaw J, Smith AH. 2009. Low-level population exposure to inorganic arsenic in the United States and diabetes mellitus: A reanalysis. Epidemiology 20(6): 807-815.
